# Association between toothbrushing and cardiovascular risk factors: a cross-sectional study using Korean National Health and Nutrition Examination Survey 2015–2017

**DOI:** 10.1186/s12903-023-03775-5

**Published:** 2024-01-02

**Authors:** Mi-Gil Moon, Si-Hyuck Kang, Sun-Hwa Kim, Shin-Young Park, Yang-Jo Seol, Chang-Hwan Yoon, Hyo-Jung Lee, Tae-Jin Youn, In-Ho Chae, Yago Leira, Eva Munoz-Aguilera, Francesco D’Aiuto

**Affiliations:** 1 Cardiovascular Center, Seoul National University Bundang Hospital, Seongnam-si, Korea; 2 Department of Internal Medicine, Seoul National University, Seoul, Korea; 3 Program of Clinical Dental Education and Dental Research Institute, School of Dentistry, Seoul National University, 101 Daehak-ro, Jongno-gu, Seoul, 03080 Korea; 4 Pre-doctoral treatment center, Seoul National University Dental Hospital, Seoul, Korea; 5 Department of Periodontology and Dental Research Institute, School of Dentistry, Seoul National University, Seoul, Korea; 6 Department of Periodontology, Section of Dentistry, Seoul National University Bundang Hospital, Seongnam-si, Korea; 7 UCL Eastman Dental Institute, Periodontology Unit, University College London, London, UK

**Keywords:** Oral hygiene, Lifestyle, Cardiovascular disease, Inflammation, Epidemiology

## Abstract

**Background:**

Previous studies have suggested that frequent toothbrushing is associated with a lower risk of future cardiovascular events. We sought to investigate further the relationship between toothbrushing, cardiovascular risk factors, and lifestyle behaviours.

**Methods:**

We analysed a cross-sectional survey including 13,761 adults aged 30 years or older without a history of cardiovascular diseases from the Korean National Health and Nutritional Examination Survey. Conventional cardiovascular risk factors (blood pressure, lipid profiles, and fasting glucose), and inflammatory markers (high-sensitivity C-reactive protein [hsCRP], and white blood cell counts [WBC]) were investigated in relation to the frequency of toothbrushing.

**Results:**

The estimated 10-year atherosclerotic cardiovascular disease (ASCVD) risk, calculated using the pooled cohort equations was 13.7%, 9.1%, and 7.3% for participants who reported toothbrushing 0–1, 2, and ≥ 3 times a day, respectively. Both conventional risk factors and inflammatory markers were significantly associated with frequent toothbrushing. However, after adjusting potential confounding factors such as age, sex, comorbidities, and lifestyle behaviours, only inflammatory markers were remained as significant factors.

**Conclusions:**

Oral hygiene behaviours are closely linked to cardiovascular risk factors. This study suggests that reduced systemic inflammatory burden may explain the benefit of improved oral hygiene in terms of cardiovascular risk.

## Introduction

Oral health and its potential link to cardiovascular disease have been investigated in recent years. [1] Cardiovascular diseases, significant global health burdens, contributes to many morbidity and mortality worldwide. [2] While traditional risk factors like high blood pressure, high cholesterol, smoking, and lack of physical activity are well-established in cardiovascular studies, the potential role of oral health in cardiovascular disease has also been supported by recent emerging evidence. [3, 4].

Several studies have suggested a positive relationship between poor oral health status and atherosclerotic cardiovascular disease [5–7]. Poor oral hygiene means a lot of deposits of dental plaques composed of oral microbiome and food debris in the oral cavity. The accumulation of dental plaques causes mild inflammation around teeth, leading to periodontal disease (gum disease) and tooth loss. When the episode of local inflammatory response is repeated, systemic inflammatory response is also triggered, contributing to the development of atherosclerosis, a key underlying process in cardiovascular diseases. [8]

However, it’s essential to note that the relationship between oral health and cardiovascular diseases is complex and multifaceted. The existing body of research in this area has yielded various findings, some supporting this association while others suggesting a more nuanced and intricate relationship. [9–11] These controversies could be explained by the fact that oral and cardiovascular diseases share common risk factors, such as advanced age, diabetes, and cigarette smoking. Additionally, the evidence suggesting that periodontal treatment induces a substantial change in cardiovascular risk or outcomes is limited. [12, 13] As a result, this topic remains a subject of ongoing investigation and debate.

Recently, observational studies have proposed that improved oral hygiene care may reduce the risk of future cardiovascular events [14, 15]. Frequent toothbrushing and regular professional cleaning were shown to be related to a reduced cardiovascular risk. However, the association is also subject to the criticism that potential confounding factors might not have been fully assessed or controlled. Individuals with better oral care habits may also have better cardiovascular behaviours. They have a higher socioeconomic status, tend to be non-smokers and physically active, and have a lower prevalence of comorbidities such as hypertension, diabetes, and obesity [15–17].

In this study, we aimed at exploring the association between cardiovascular risk and frequency of toothbrushing in the context of traditional risk factors and inflammatory markers, using a nationally representative cross-sectional cohort of the Korean population. We sought to identify potential confounders between toothbrushing and cardiovascular risk, and to estimate the impact of toothbrushing independent of the confounding factors.

## Methods

### Study design, population and cohort

**Fig. 1:**
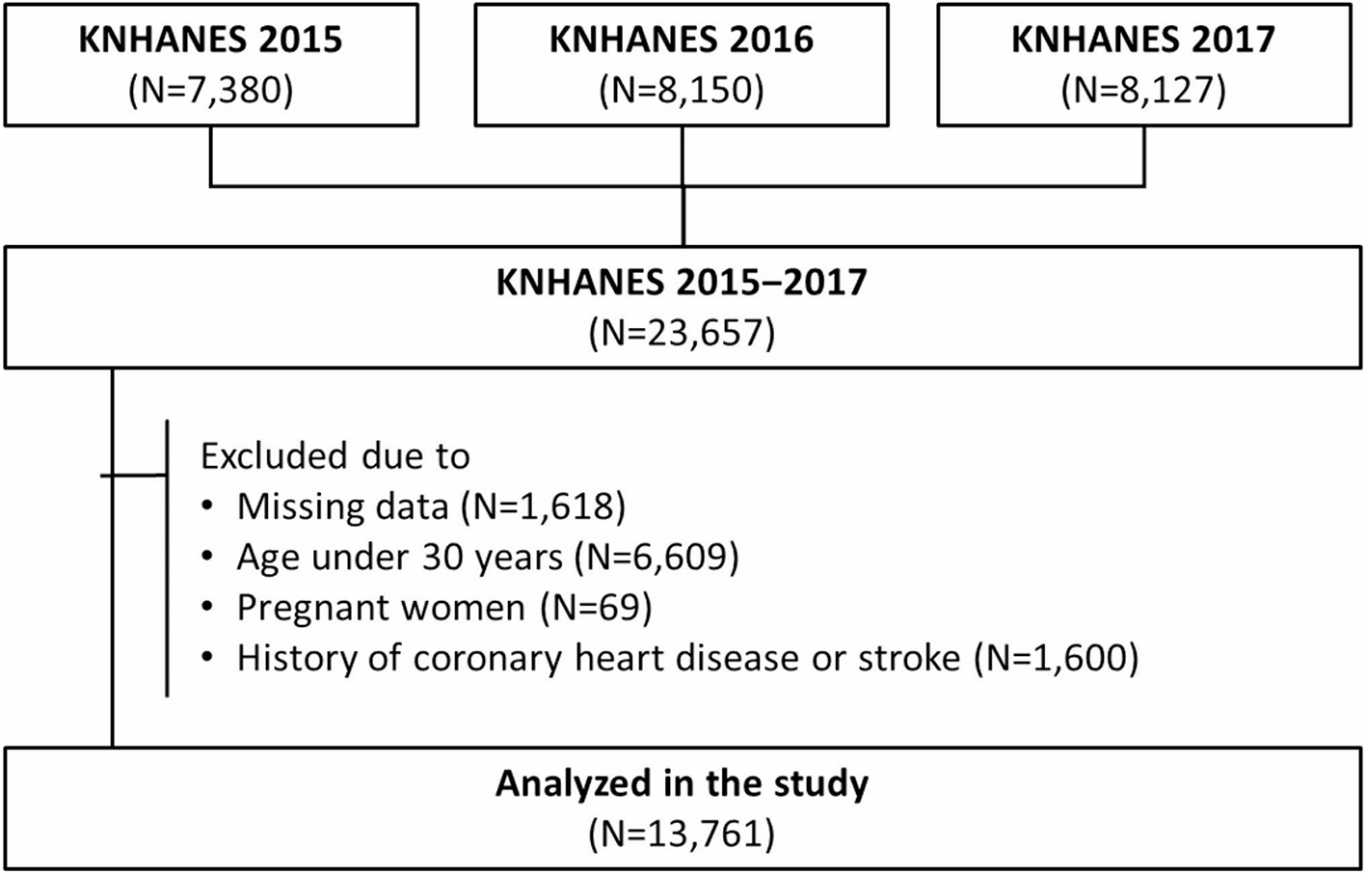
Description of the study population

This was a cross-sectional study using data from the Korean National Health and Nutritional Examination Survey (KNHANES), a nationwide survey, conducted from 2015 to 2017. Of the 23,657 participants, individuals less than 30 years, pregnant women, those who had a history of coronary heart disease or stroke, and those with missing data were excluded (Fig. 1). Thus, data of a total of 13,761 participants were analyzed in the study. Information on the participants’ demographic characteristics, socioeconomic status, health-related behaviours, healthcare utilization, biochemical and clinical profiles for non-communicable diseases, and dietary intakes are included in the KNHANES [18]. This study conformed to the principles outlined in the Declaration of Helsinki and guidelines of Strengthening the Reporting of Observational Studies in Epidemiology (STROBE). This study was approved by the Institutional Review Board (IRB) of Seoul National University Bundang Hospital (X-1910-572-902), and the requirement for informed consent was waived because of the retrospective nature of the study and minimal expected risk to the participants.

### Definition and measurements in KNHANES data

Participants were asked to report on their subjective health behaviours using self-report questionnaires (11). The frequency of toothbrushing in a day was considered the primary independent variable. The survey participants were divided into three groups: ≥3 times/day, 2 times/day, and 0−1 times/day. Data on health-related lifestyle behaviours such as smoking status, the consumption of alcohol, and regular leisure-time physical activity were also collected.

Blood pressure (BP) was measured by trained nurses using mercury sphygmomanometers. BP was measured three times on the participants’ right arms after resting in a seated position for at least 5 min. Anthropometric measurements included body weight, height, and waist circumference. Laboratory tests included total cholesterol (mg/dL), low density lipoprotein (LDL)-cholesterol (mg/dL), high density lipoprotein (HDL)-cholesterol (mg/dL), triglyceride (mg/dL), fasting plasma glucose (mg/dL), high-sensitivity C-reactive protein (hsCRP, mg/L), and white blood cell (WBC, 10³/µL) counts.

Comorbidities such as hypertension, dyslipidaemia, and diabetes, as well as their management status were assessed. Hypertension was defined either as systolic BP ≥ 140 mmHg, diastolic BP ≥ 90 mmHg, or the use of an antihypertensive medication [19]. Awareness of hypertension was defined as having had a medical diagnosis of hypertension by medical personnel. Treatment was defined as taking antihypertensive medications for more than 20 days per month. Adequate hypertension control was defined as having systolic and diastolic BP of < 140/90 mmHg. Dyslipidaemia was defined either as total cholesterol ≥ 240 mg/dL, triglyceride ≥ 200 mg/dL, or the use of lipid-lowering therapy. Diabetes was defined as fasting plasma glucose ≥ 126 mg/dL, or treatment with oral hypoglycaemic agents or insulin.

### Dependent variables: cardiovascular risk factors

Dependent study variables included conventional cardiovascular risk factors, the estimated 10-year risk for a first atherosclerotic cardiovascular disease (ASCVD) event, and inflammatory markers. Conventional risk factors were systolic and diastolic BP, total cholesterol, HDL-cholesterol, non-HDL-cholesterol, triglycerides, LDL-cholesterol, and fasting plasma glucose. The estimated 10-year ASCVD risk was calculated using the pooled cohort equations including variables such as age, sex, race, total cholesterol, HDL-cholesterol, systolic BP, hypertension treatment status, diabetes, and smoking status [20]. Estimates for White races were used in this study because Asian-specific values were not available for the equations. Inflammatory markers included hsCRP, and WBC levels.

### Statistical analysis

Since the KNHANES used a complex survey design, statistical analyses were performed considering the weight of each sample. The weights were derived for sample participants to represent the Korean population by accounting for the survey design, survey non-response, and post-stratification. Baseline characteristics were reported as means ± standard errors for continuous variables and as percentages (standard error) for dichotomous variables. Generalized linear models were used to evaluate the relationships between the independent and dependent variables. Multivariable-adjustment models were developed as follows: model one was unadjusted; model two was adjusted for age and sex; model three was adjusted for age, sex, and lifestyle behaviours such as obesity, current smoking, heavy alcohol drinking, and physical activity; model four was adjusted for the variables listed above as well as hypertension, diabetes, and dyslipidaemia. Since the 10-year ASCVD risk was not normally distributed, it was log-transformed in the statistical model. Statistical analyses were conducted using R programming version 3.3.0 (http://www.R-project.org; The R Foundation for Statistical Computing, Vienna, Austria). *P*-values less than 0.05 were considered statistically significant.

## Results

Among the total study population, 10.4%, 37.2%, and 52.4% brushed their teeth 0−1, 2, and ≥ 3 times a day, respectively. Participants who brushed their teeth more frequently were younger and predominantly female (Table 1). They had a lower prevalence of comorbidities such as hypertension, diabetes, and dyslipidaemia. Improved oral hygiene was also linked to better cardiovascular lifestyle behaviours such as lower frequencies of cigarette smoking and alcohol drinking, higher amount of physical activity, lower body mass indexes and waist circumferences, and higher potassium intake. The total caloric intake was similar across the groups, whilst sodium intake increased with improved health behaviours. The management status of hypertension had an inverse relationship with toothbrushing frequency. Those with frequent toothbrushing showed significantly lower rates of awareness, treatment, and control of hypertension. Treatment of diabetes and dyslipidaemia were not significantly associated with toothbrushing frequency.

**Table 1:** Baseline characteristics according to toothbrushing behaviours

	Total	Tooth brushing			
		≥ 3 times/day	2 times/day	0−1 time/day	P
N (proportion) – million	29.9 (100)	15.7 (52.4)	11.1 (37.2)	3.1 (10.4)	
**Demographics**					
Age − years	51.3 ± 0.2	48.8 ± 0.2	52.7 ± 0.3	58.3 ± 0.5	< 0.001
Male sex − %	48.3 ± 0.4	44.2 ± 0.6	49.1 ± 0.8	65.8 ± 1.3	< 0.001
**Lifestyle behaviors**					
Current smoker − %	20.9 ± 0.5	16.7 ± 0.6	24.1 ± 0.8	30.5 ± 1.5	< 0.001
Regular physical activity − %	45.3 ± 0.6	50.2 ± 0.8	42.3 ± 0.9	35.5 ± 1.4	< 0.001
Body mass index – kg/m^2^	24.1 ± 0.0	23.8 ± 0.0	24.4 ± 0.1	24.5 ± 0.1	< 0.001
Waist circumference – cm	83.1 ± 0.1	81.8 ± 0.1	84.1 ± 0.2	86.0 ± 0.3	< 0.001
Obesity − %	31.9 ± 0.6	30.2 ± 0.7	32.7 ± 0.9	37.7 ± 1.6	< 0.001
Drinking − %					< 0.001
Complete or near abstinence	36.2 ± 0.5	36.1 ± 0.7	36.2 ± 0.8	36.6 ± 1.5	
Moderate consumption	31.5 ± 0.5	33.7 ± 0.7	30.7 ± 0.8	22.4 ± 1.4	
Heavy drinking	32.3 ± 0.5	30.2 ± 0.7	33.1 ± 0.9	41.1 ± 1.6	
Diet					
Calorie intake − kcal/day	2,000 ± 12	2,015 ± 15	1,991 ± 19	1,949 ± 33	0.056
Sodium intake − mg/day	3,850 ± 36	3,906 ± 51	3,823 ± 47	3,660 ± 88	0.018
Potassium intake − mg/day	3,048 ± 22	3,147 ± 26	2,993 ± 33	2,738 ± 56	< 0.001
**Comorbidities** − %					
Hypertension	30.6 ± 0.5	25.4 ± 0.6	34.6 ± 0.8	42.8 ± 1.5	< 0.001
Diabetes	11.0 ± 0.3	8.2 ± 0.4	12.9 ± 0.6	19.3 ± 1.3	< 0.001
Dyslipidemia	21.4 ± 0.4	19.9 ± 0.5	22.9 ± 0.7	23.2 ± 1.4	0.002
**Management of comorbidities** − %					
Hypertension awareness	67.6 ± 0.9	63.5 ± 1.3	68.7 ± 1.4	76.4 ± 2.0	< 0.001
Hypertension treatment	63.6 ± 0.9	59.4 ± 1.4	65.0 ± 1.4	71.9 ± 2.1	< 0.001
Hypertension control	47.0 ± 1.0	44.6 ± 1.3	47.8 ± 1.4	51.7 ± 2.3	0.017
Diabetes treatment	94.0 ± 0.9	93.6 ± 1.3	93.3 ± 1.4	96.4 ± 1.6	0.296
Dyslipidemia treatment	60.3 ± 1.1	57.4 ± 1.7	62.7 ± 1.8	63.4 ± 3.4	0.080

The Chi-square test was performed for categorical variables and analysis of variance test was used for continuous variables

When assessing the relationship between dental hygiene habits and oral health status, frequent toothbrushing was associated with a lower prevalence of discomfort during chewing (Table 2). Those who frequently brushed their teeth made frequent dental visits and had favourable oral hygiene behaviours such as floss and interdental brush use. Study participants with better oral hygiene behaviours had favourable cardiovascular risk profiles. Those who reported more frequent toothbrushing had lower systolic BP and lower non-HDL cholesterol, triglyceride, and fasting plasma glucose levels. Additionally, they had higher HDL cholesterol levels. LDL cholesterol levels did not differ significantly. The estimated 10-year ASCVD risk was 13.7%, 9.1%, and 7.3% for those who brushed their teeth 0–1, 2, and ≥ 3 times a day, respectively.

**Table 2:** Oral health status and cardiovascular risk factors according to toothbrushing behaviours

	Total	Tooth brushing			
		≥ 3 times/day	2 times/day	0−1 time/day	P
**Oral status**					
**Behaviors related to dental problems**					
Discomfort during chewing -%	21.9 ± 0.5	17.4 ± 0.6	24.4 ± 0.7	35.9 ± 1.5	< 0.001
Dental visit −%					
Dental visits for any reasons	55.4 ± 0.6	59.8 ± 0.7	52.8 ± 0.8	43.0 ± 1.6	< 0.001
≥ 1 time/yr for regular check-up only	35.5 ± 0.7	42.5 ± 0.8	29.7 ± 0.8	20.4 ± 1.3	< 0.001
No dental visit despite dental problems	28.3 ± 0.5	25.6 ± 0.6	30.8 ± 0.8	32.5 ± 1.5	< 0.001
**Oral hygiene behavior**					
Floss or interdental brush – %	37.0 ± 0.6	44.4 ± 0.8	31.9 ± 0.8	17.6 ± 1.2	< 0.001
Professional cleaning – %	31.0 ± 0.5	35.8 ± 0.7	28.2 ± 0.7	17.2 ± 1.2	< 0.001
**Cardiovascular risk factors**					
Systolic blood pressure − mmHg	118.7 ± 0.2	117.0 ± 0.2	119.7 ± 0.3	123.5 ± 0.5	< 0.001
Diastolic blood pressure − mmHg	76.4 ± 0.1	76.3 ± 0.2	76.7 ± 0.2	76.4 ± 0.3	0.338
Total cholesterol − mg/dL	196.0 ± 0.4	196.3 ± 0.5	196.1 ± 0.6	194.3 ± 1.2	0.169
HDL-cholesterol − mg/dL	50.7 ± 0.1	51.8 ± 0.2	50.1 ± 0.2	47.3 ± 0.4	< 0.001
Non-HDL-cholesterol − mg/dL	145.3 ± 0.4	144.6 ± 0.5	146.0 ± 0.6	147.1 ± 1.1	0.016
Triglyceride − mg/dL	146.0 ± 1.6	137.2 ± 1.8	152.3 ± 2.9	168.7 ± 5.5	< 0.001
LDL cholesterol − mg/dL	118.7 ± 0.3	119.2 ± 0.4	118.5 ± 0.6	117.3 ± 1.0	0.073
Fasting plasma glucose – mg/dL	101.6 ± 0.3	99.2 ± 0.3	102.8 ± 0.4	109.0 ± 1.1	< 0.001
hsCRP* – mg/L	1.2 ± 0.0	1.1 ± 0.0	1.3 ± 0.0	1.6 ± 0.1	< 0.001
White blood cell count − 10³/µL	6.3 ± 0.0	6.2 ± 0.0	6.4 ± 0.0	6.7 ± 0.1	< 0.001

The Chi-square test was performed for categorical variables and analysis of variance test was used for continuous variables. *Abbreviations*: hsCRP, high-sensitivity C-reactive protein

**Fig. 2:**
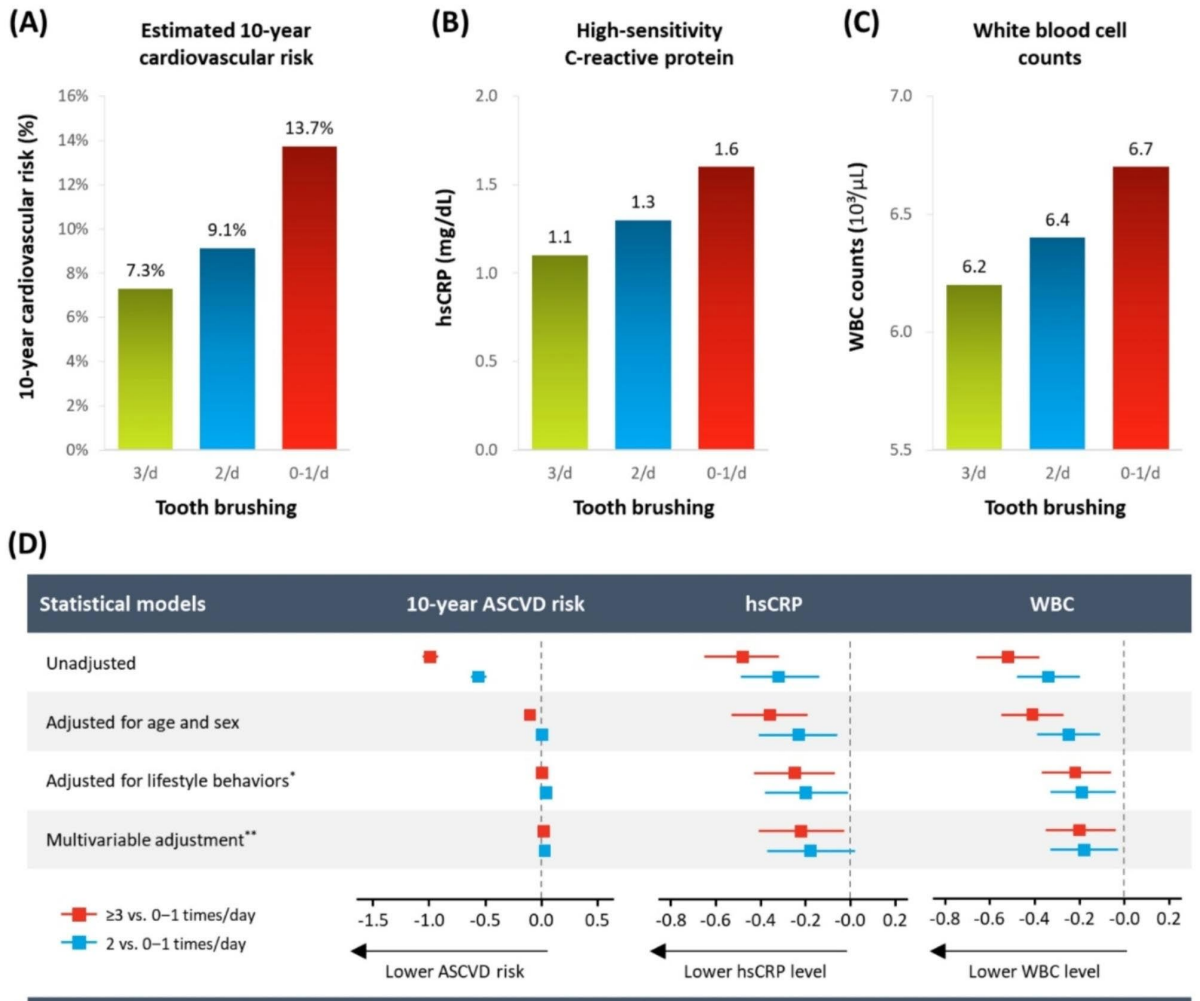
Unadjusted cardiovascular risk profiles according to toothbrushing frequency. (**A**) Estimated 10-year atherosclerotic cardiovascular risk calculated using pooled cohort equations, (**B**) high-sensitivity C-reactive protein (hsCRP), and (**C**) white blood cell (WBC) counts. Multivariable adjustment for the association between toothbrushing frequency and cardiovascular risk profiles (**D**). The estimated 10-year ASCVD risk was log-transformed. The red box and line indicate a comparison of toothbrushing ≥ 3 vs. 0−1 times/day; blue indicates that of 2 vs. 0−1 times/day. ^*^Adjusted for age, sex, obesity, current smoking, heavy alcohol drinking, and physical activity; ^**^Adjusted for age, sex, obesity, current smoking, heavy alcohol drinking, physical activity, hypertension, diabetes, and dyslipidaemia. *Abbreviations*: hsCRP, high-sensitivity C-reactive protein; WBC, white blood cell

The differences in traditional cardiovascular risk factors were mitigated after adjusting for potential confounders (Table 3). For example, the differences in the estimated 10-year ASCVD risk based on toothbrushing behaviours were largely attributable to age and sex (≥ 3 vs. 0−1 times/day: unadjusted P < 0.001, age-sex adjusted P < 0.001; 2 vs. 0−1 times/day: unadjusted P < 0.001, age-sex adjusted P = 0.517). The association further lost statistical significance after adding lifestyle behaviours such as obesity, current smoking, heavy alcohol drinking, and physical activity into the model. (≥ 3 vs. 0−1 times/day: lifestyle adjusted P = 0.755; 2 vs. 0−1 times/day: lifestyle adjusted P = 0.068) The estimate became neutral after including comorbidities such as hypertension, diabetes, and dyslipidaemia into the model (≥ 3 vs. 0−1 times/day: multivariable adjusted P = 0.301; 2 vs. 0−1 times/day: lifestyle adjusted P = 0.066). Other conventional risk factors such as systolic BP, non-HDL-cholesterol, HDL-cholesterol, triglyceride, and fasting glucose showed similar patterns. (Fig. 2)

**Table 3:** Multivariable adjustment for the relationship between tooth brushing and cardiovascular risk factors

	Tooth brushing			
	≥ 3 vs. 0−1 times/day		2 vs. 0−1 times/day	
	Difference (95% CI)	P	Difference (95% CI)	P
**Log (10-yr ASCVD risk) – logit link**				
Unadjusted	-0.99 (-1.06, -0.92)	< 0.001	-0.56 (-0.63, -0.49)	< 0.001
Adjusted for age and sex	-0.10 (-0.14, -0.06)	< 0.001	0.01 (-0.03, 0.06)	0.517
Adjusted for lifestyle behaviors^*^	0.01 (-0.04, 0.05)	0.755	0.04 (0.00, 0.09)	0.068
Multivariable adjustment^**^	0.02 (-0.02, 0.05)	0.301	0.03 (0.00, 0.07)	0.066
**Systolic blood pressure–mmHg**				
Unadjusted	-6.44 (-7.45, -5.43)	< 0.001	-3.78 (-4.78, -2.78)	< 0.001
Adjusted for age and sex	-0.89 (-1.88, 0.11)	0.082	-0.29 (-1.27, 0.70)	0.569
Adjusted for lifestyle behaviors^*^	-0.59 (-1.64, 0.45)	0.266	-0.44 (-1.46, 0.59)	0.403
Multivariable adjustment^**^	-0.23 (-1.21, 0.74)	0.639	-0.40 (-1.36, 0.57)	0.418
**Diastolic blood pressure–mmHg**				
Unadjusted	-0.07 (-0.77, 0.62)	0.833	0.27 (-0.46, 1.01)	0.466
Adjusted for age and sex	0.53 (-0.15, 1.20)	0.125	0.83 (0.11, 1.54)	0.023
Adjusted for lifestyle behaviors^*^	1.01 (0.32, 1.71)	0.005	0.85 (0.13, 1.58)	0.021
Multivariable adjustment^**^	1.03 (0.34, 1.71)	0.003	0.80 (0.09, 1.50)	0.027
**Total cholesterol–mg/dL**				
Unadjusted	2.00 (-0.42, 4.42)	0.105	1.73 (-0.75, 4.22)	0.172
Adjusted for age and sex	1.47 (-1.04, 3.97)	0.252	1.39 (-0.1.12, 3.90)	0.280
Adjusted for lifestyle behaviors^*^	2.57 (-1.13, 4.25)	0.070	1.56 (-0.20, 5.34)	0.256
Multivariable adjustment^**^	0.77 (-1.79, 3.33)	0.558	0.41 (-2.18, 3.01)	0.754
**HDL-cholesterol–mg/dL**				
Unadjusted	4.55 (3.77, 5.32)	< 0.001	2.86 (2.04, 3.68)	< 0.001
Adjusted for age and sex	1.96 (1.18, 2.73)	< 0.001	1.03 (0.21, 1.84)	0.014
Adjusted for lifestyle behaviors^*^	0.94 (0.08, 1.80)	0.033	0.67 (-0.21, 1.55)	0.134
Multivariable adjustment^**^	0.66 (-0.23, 1.58)	0.145	0.56 (-0.33, 1.46)	0.219
**Non-HDL-cholesterol–mg/dL**				
Unadjusted	-2.54 (-4.95, -0.13)	0.040	-1.14 (-3.63, 1.34)	0.368
Adjusted for age and sex	-0.48 (-2.98, 2.01)	0.703	0.34 (-2.14, 2.86)	0.789
Adjusted for lifestyle behaviors^*^	1.63 (-1.06, 4.32)	0.235	0.87 (-1.79, 3.52)	0.522
Multivariable adjustment^**^	0.10 (-2.37, 2.58)	0.934	-0.16 (-2.70, 2.38)	0.903
**Triglyceride–mg/dL**				
Unadjusted	-31.50 (-42.56, -20.44)	< 0.001	-16.39 (-28.29, -4.48)	0.007
Adjusted for age and sex	-19.31 (-30.77, -7.85)	0.001	-6.98 (-19.00, 5.03)	0.255
Adjusted for lifestyle behaviors^*^	-7.98 (-18.68, 2.72)	0.144	-2.39 (-13.76, 8.99)	0.681
Multivariable adjustment^**^	-8.26 (-19.13, 2.60)	0.137	-2.98 (-14.52, 8.56)	0.612
**LDL cholesterol–mg/dL**				
Unadjusted	1.29 (-1.93, 4.51)	0.433	2.59 (-0.82, 6.01)	0.137
Adjusted for age and sex	0.95 (-2.36, 4.27)	0.573	2.42 (-1.00, 5.85)	0.165
Adjusted for lifestyle behaviors^*^	1.24 (-2.34, 4.82)	0.499	1.85 (-1.70, 5.40)	0.309
Multivariable adjustment^**^	0.07 (-3.43, 3.57)	0.968	0.52 (-2.98, 4.03)	0.770
**Fasting plasma glucose–mg/dL**				
Unadjusted	-9.82 (-12.10, -7.54)	< 0.001	-6.20 (-8.52, -3.89)	< 0.001
Adjusted for age and sex	-5.49 (-7.92, -3.05)	< 0.001	-3.38 (-5.79, -0.97)	0.006
Adjusted for lifestyle behaviors^*^	-4.70 (-7.26, -2.14)	< 0.001	-3.04 (-5.57, -0.51)	0.191
Multivariable adjustment^**^	-1.53 (-3.23, 0.16)	0.076	-1.18 (-2.88, 0.52)	0.175
hsCRP*–mg/L				
Unadjusted	-0.48 (-0.65, -0.32)	< 0.001	-0.32 (-0.49, -0.14)	< 0.001
Adjusted for age and sex	-0.36 (-0.53, -0.19)	< 0.001	-0.23 (-0.41, -0.06)	0.010
Adjusted for lifestyle behaviors^*^	-0.25 (-0.43, -0.07)	0.007	-0.20 (-0.38, -0.01)	0.041
Multivariable adjustment^**^	-0.22 (-0.41, -0.03)	0.022	-0.18 (-0.37, 0.02)	0.075
**White blood cell–10³/µL**				
Unadjusted	-0.52 (-0.66, -0.38)	< 0.001	-0.34 (-0.48, -0.20)	< 0.001
Adjusted for age and sex	-0.41 (-0.55, -0.27)	< 0.001	-0.25 (-0.39, -0.11)	< 0.001
Adjusted for lifestyle behaviors^*^	-0.22 (-0.37, -0.06)	0.005	-0.19 (-0.33, -0.04)	0.016
Multivariable adjustment^**^	-0.20 (-0.35, -0.04)	0.013	-0.18 (-0.33, -0.03)	0.021

Generalized linear models were used to evaluate the relationships between the independent and dependent variables. Estimated 10-year ASCVD risk was log-transformed. ^*^Adjusted for age, sex, obesity, current smoking, heavy alcohol drinking, and physical activity; and ^**^Adjusted for age, sex, hypertension, diabetes, and dyslipidemia, obesity, current smoking, heavy alcohol drinking, and physical activity. *Abbreviations*: hsCRP, high-sensitivity C-reactive protein; WBC, white blood cell

Notably, hsCRP and WBC levels consistently showed significant associations with toothbrushing frequency even after adjusting for the confounding factors. Participants who reported brushing their teeth ≥ 3 times/day had 0.48 mg/L and 0.32 mg/L lower levels of hsCRP than those who brushed their teeth 2 times/day and 0−1 time/day, respectively (unadjusted P-values < 0.001 for both). Although being partially attenuated, the relationship remained statistically significant after adjusting for all the variables mentioned above between participants brushing their teeth ≥ 3 vs. 0−1 times/day. (≥ 3 vs. 0−1 times/day: -0.22 mg/L, P = 0.022; 2 vs. 0−1 times/day: -0.18 mg/L, P = 0.075). The WBC levels similarly showed significant differences according to toothbrushing behaviours after multivariable adjustment. (-0.20 × 10³/µL, P = 0.013; and − 0.18 × 10³/µL, P = 0.021, respectively).

## Discussion

In the current study participants who brushed their teeth more frequently had lower cardiovascular risk including lower systolic BP, higher HDL-cholesterol, and lower 10-year ASCVD risk calculated using the pooled cohort equations. They also had lower systemic inflammation as assessed by serum hsCRP and WBC levels. This study also confirmed that the differences in cardiovascular risk based on toothbrushing habits were heavily confounded by age, sex, comorbidities, and lifestyle factors. However, the association between inflammatory marker and toothbrushing behaviour were independent of common confounding factors.

The association between periodontal and cardiovascular diseases has long been debated [3, 4, 21]. A number of studies have demonstrated a close link between the two diseases [5–7, 22]; however, a causal relationship has yet to be established [3]. The two diseases share common risk factors such as smoking, diabetes, and advanced age [15, 23]. The associations between the two diseases disappear or are largely attenuated after controlling for confounding factors. There is also limited evidence supporting the benefit of periodontal treatment in reducing cardiovascular risk. Indeed, recent evidence from randomized controlled trials demonstrated that periodontal treatment reduces systemic levels of interleukin (IL)-6, C-reactive protein, BP, total cholesterol, and E-selectin [12, 24], improves endothelial function [12], and improve metabolic helps glycaemic control and kidney function in patients with type 2 diabetes [25].

Recent observational studies have suggested that improved oral hygiene care may lead to better cardiovascular outcomes. De Oliveira and co-workers analysed 11,869 adults with an average of 8.1 years of follow-up from the Scottish Health Survey showing that participants with poor oral hygiene (never/rarely brushed their teeth) had a 70% increased risk of a cardiovascular event [14]. A study by Park and co-workers, including 247,696 Korean adults who were followed for a mean of 9.5 years, found that performing one more toothbrushing a day and regular dental visits for professional cleaning were associated with a 9% and 14% lower risk of cardiovascular events, respectively [15].

The observations that improving oral health reduces cardiovascular risk add to the causal relationship between oral health and cardiovascular diseases. However, the relationship is also not free from confounding effects. As shown in this study, participants who brush their teeth frequently have favourable cardiovascular lifestyle behaviours such as no cigarette smoking, high physical activity, and a healthy diet [26]. We confirmed that the associations of toothbrushing frequency with conventional cardiovascular risk factors such as systolic BP, total cholesterol, HDL-cholesterol, and fasting glucose were largely attributable to the confounding effects.

The association of oral hygiene with systemic inflammation persisted after controlling for potential confounding factors. hsCRP is one of the most extensively studied biomarkers in predicting cardiovascular risk estimation [4]. While conventional cardiovascular risk factors such as age, sex, BP, blood cholesterol, and cigarettes smoking account for 70−80% of the future risk of cardiovascular events, there is still a remaining uncertainty of 20−30% [27, 28]. Robust evidence indicate that inflammation plays an additional and critical role in atherothrombosis [29, 30]. hsCRP, a downstream biomarker for inflammation, was shown to be associated with an increased risk of cardiovascular events [31, 32]. Diseases characterized by a chronic inflammatory exposure such as rheumatoid arthritis have been associated with increased risk of cardiovascular events [33].

Oral hygiene care is directly linked to the quantity of the dental biofilm and quality of oral microbiome. Daily toothbrushing, with or without adjunctive use of flossing and interdental brush reduces oral microbial burden, which prevents oral diseases such as dental caries and periodontal diseases [34]. Periodontitis is a chronic inflammatory disease resulting from a dysbiotic dental biofilm harbouring pathogenic gram-negative bacteria such as *Porphyromonas gingivalis*. The presence of a key pathogen such as *P. gingivalis* determine deregulated immune and inflammatory reaction to protect the host from bacterial invasion [35]. However, as pathogenic bacteria grow, activation of inflammatory responses may become chronic and have the potential to induce a host response. There is a wealth of evidence confirming that patients with periodontitis exhibit higher levels of systemic inflammation compared to controls and that periodontal treatment normalize/reduce the inflammatory burden [36]. As inflammation is thought to be one of the contributors to the pathogenesis of vascular diseases [37], the link between periodontitis and vascular health could be driven by an altered body response to the dental biofilm. *P. gingivalis* is able to activate endothelial cells to express IL-6, IL-8, and vascular cell adhesion molecule 1, which ultimately could lead to systemic inflammation [38, 39]. Experimental animal studies have shown induction of atherosclerosis by *P. gingivalis* [40].

The greatest limitation of the present study is its observational and cross-sectional nature. Findings of this study should be considered hypothesis-generating. The benefit of oral hygiene care can only be confirmed by prospective randomized controlled trials. Secondly, data on oral hygiene behaviours were collected using self-report questionnaire, which may be prone to recall bias. Thirdly, although we extracted various potential confounders, we cannot rule out the presence of unmeasured confounding factors. Lastly, the pooled cohort equations used in estimated 10-year ASCVD risk have been developed for White and African American races and not been fully validated in East Asian ethnicities [41].

## Conclusions

This cross-sectional study confirmed that individuals with better oral hygiene care had favourable cardiovascular risk profiles. They also had favourable demographics characteristics and cardiovascular lifestyle behaviours. The associations between conventional risk factors and toothbrushing were mostly accounted for by confounding factors. Nevertheless, the associations with inflammatory markers including hsCRP and WBC levels were found to be independent of the confounding effects. Our findings suggest that decreased systemic inflammation might be the key mechanism of lower cardiovascular risk associated with frequent toothbrushing.

## Data Availability

KNHANES data can be accessed and downloaded from the KNHANES homepage (URL: https://knhanes.cdc.go.kr/knhanes/eng/index.do).

## List of abbreviations

ASCVD: Atherosclerotic cardiovascular disease
BP: Blood pressure
HDL: high density lipoprotein
hsCRP: high-sensitivity C-reactive protein
KNHANES: Korea National Health and Nutrition Examination Survey
LDL: low density lipoprotein
WBC: white blood cell counts
